# Comparative analysis on Facebook post interaction using DNN, ELM and LSTM

**DOI:** 10.1371/journal.pone.0224452

**Published:** 2019-11-12

**Authors:** Sabih Ahmad Khan, Hsien-Tsung Chang

**Affiliations:** 1 Department of Computer Science and Information Engineering, Chang Gung University, Taoyuan 33302, Taiwan; 2 Department of Physical Medicine and Rehabilitation, Chang Gung Memorial Hospital, Taoyuan 33302, Taiwan; Politechnika Krakowska im Tadeusza Kosciuszki, POLAND

## Abstract

This study presents a novel research approach to predict user interaction for social media post using machine learning algorithms. The posts are converted to vector form using word2vec and doc2vec model. These two methods are used to analyse the best approach for generating word embeddings. The generated word embeddings of post combined with other attributes like post published time, type of post and total interactions are used to train machine learning algorithms. Deep neural network (DNN), Extreme Learning Machine (ELM) and Long Short-Term Memory (LSTM) are used to compare the prediction of total interaction for a particular post. For word2vec, the word vectors are created using both continuous bag-of-words (CBOW) and skip-gram models. Also the pre-trained word vectors provided by google is used for the analysis. For doc2vec, the word embeddings are created using both the Distributed Memory model of Paragraph Vectors (PV-DM) and Distributed Bag of Words model of Paragraph Vectors (PV-DBOW). A word embedding is also created using PV-DBOW combined with skip-gram.

## 1 Introduction

Social media has become an important part of people’s lives. It helps in creating content and sharing information among virtual communities and networks. Users spend a lot of time on social networking sites (SNS) like Facebook, LinkedIn and Twitter to interact with each other. Organizations have also understood the potential of social media and are now exploiting it to promote their products and analyze customer satisfaction [1]. Researchers are also trying to understand human behavior on these platforms by adopting different strategies like viral product design [2], information diffusion model [3], network diffusion [4] and user influence [5]–[7]. Facebook is an important platform for a company to build their brand and reach out to a large customer base. As of September 2017, Facebook stands first for the number of active users on any SNS [8] with 2,072 million monthly active users (MAUs) [9] and is still growing.

In this era of huge competition between a company and its rivals, influencing a customer is a big challenge. The presence of these competitions are also visible on SNS, so companies have integrated social media as an additional marketing channel [10] along with traditional ones such as news and television media [11]. Traditional media was lacking user participation so the customer satisfaction was unpredictable but social media brought two-way communication where buyer and seller can interact with each other and it can create a long lasting relationship between a company and its customer [12]. Recognizing the power of SNS, many companies have created their brand pages on Facebook to advertise their products using persuasive messages [13] and increase brand post popularity [14], [15]. The brand post popularity [16] is dependent on various factors [17] like vividness, interactivity and post content [14] so it will be highly beneficial for brand managers to know the impact of post beforehand in order to make a sound decision [18]–[20], when and what type of post should be published.

This problem of post impact prediction acted as our motivation and in our proposed work we have tried to solve this problem using machine learning algorithms. We have done comparative analysis on Deep neural network (DNN), Extreme Learning Machine (ELM) [21] and Long Short-Term Memory (LSTM) [22], [23] using natural language processing (NLP) techniques like word vectors (word2vec) [24] and paragraph vectors (doc2vec) [25] to predict impact of post before it is published. In word2vec model, word embeddings for each word is created with word as the target in CBOW model and context words as the target in skip-gram model [24], [26]. In doc2vec model, each paragraph or post message is mapped to a unique vector using word as the target in PV-DM model and context words as the target in PV-DBOW model [25].

**Fig 1:**
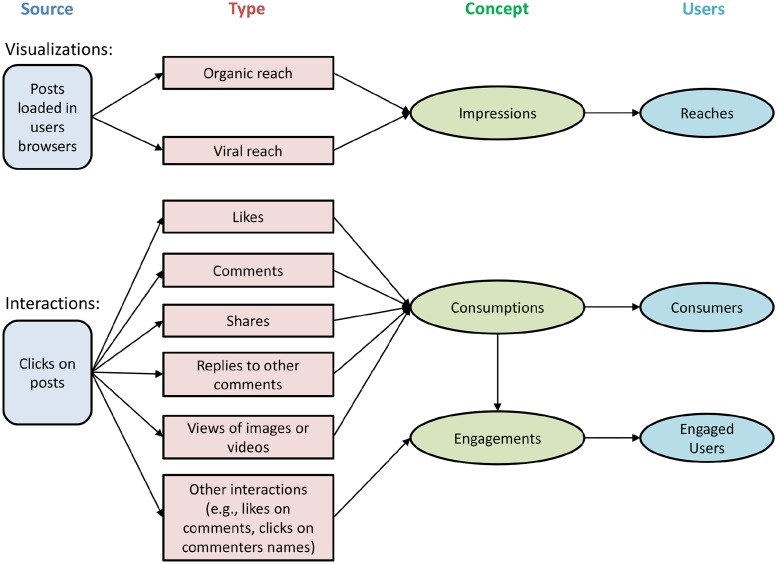
Facebook post performance conceptual map [27].

The performance of a Facebook post can be obtained through visualizations or interactions [27] as shown in Fig 1. The number of times a post is displayed on users browser either directly (organic reach) since user liked the page or through another users interaction (viral reach) comes under visualizations which provides the post impression count. The user activities such as likes, comments or shares on a post determines user interaction with the post. The user interaction is a strong measure for a post performance since it shows user attentiveness and engagement with the post while visualization only provides the information of post displayed on users browser. Considering this concept, we used Facebook post interaction for comparative analysis in our study. The goal of this work is to predict total interactions a Facebook post will receive once it is published and compare the machine learning models to find the best approximation.

The remainder of this paper is organized as follows. Section 2 reviews existing studies that are related to our work. Section 3 briefly introduces algorithms used in our analysis. Section 4 provides dataset description collected from Facebook. Section 5 explains the conducted experiments and Section 6 analyze the experimental results. Section 7 concludes this paper with future work discussion.

## 2 Related work

Recent advances in social media has attracted many researchers to study this field and provide some guidelines that can help brand managers in developing social media marketing strategy. The authors in [11] have studied the effects of Facebook post type, post category and weekday of posting on user interaction level in terms of likes, comments and interaction duration. The post type and weekday are collected through Facebook API but posts are divided into seven different categories manually. The generalized version of [11] is proposed in [1] where data is collected from 14 different brand pages of Facebook instead of a single page. This study showed evidence of increasing fan activities on various brand posts.

Moro et al. [27] utilized data mining approach to predict impact of post prior to its publication. Seven input features category, page total likes, type, month, hour, weekday and paid are selected to analyze the post performance using twelve performance metrics. Further a performance metric which is solely dependent on impact of post with least error is used to assess how the input features influenced its outcome.

In [28], the Facebook post performance for healthcare dataset is analyzed using Artificial Neural Network (ANN) and Deep Neural Network (DNN). The objective of this study was to find the best predictive model for user engagement with the post. For each model, eight input parameters were selected from Facebook post and three output units to predict if the post will have low, medium or high user engagement. The number of hidden layers and nodes in each layer are varied to find the best predictive model with highest accuracy for user engagement. This study is further extended in [29] where K nearest neighbor (KNN), Gaussian mixture model (GMM) and K-means are applied to the healthcare dataset for comparative analysis.

The authors in [30] predicted the personality traits using Facebook wall post. This study was conducted only for Chinese users where users are given a questionnaire to evaluate themselves from 1 (strongly disagree) to 5 (strongly agree) on Big Five model of personality i.e., extraversion, neuroticism, agreeableness, conscientiousness and openness. Further the score of extraversion is used for binary classification where users are classified as introverts with scores from 1 to 3 and extroverts with scores from 4 to 5. The BoW model is used for text feature extraction from Facebook wall post for each user and SVM is used as learning algorithm.

All the above mentioned works has predicted some useful information from SNS but except [30], none of them included the post message in its model input which is an important parameter since user interaction with the post mainly depend on its content. In our study we integrated post content which is converted to numeric values using two NLP techniques i.e., word2vec and doc2vec along with other attributes to predict post popularity which is the novelty of this work.

## 3 Preliminaries

Deep Neural Networks (DNN) is state-of-the-art in machine learning and has been used in many fields to solve complex problems such as object detection and speech recognition. Extreme Learning Machine (ELM) is extremely fast to train the model in comparison to DNN and has been giving tough competition to DNN since its inception. Long Short-Term Memory (LSTM) solved a major bottleneck in recurrent neural networks to learn long-range dependencies and has been widely used in natural language applications. Considering the above reasons, we have used DNN, ELM and LSTM in our study for comparative analysis in predicting Facebook post interaction.

This study integrates post message, so we have used two NLP techniques to convert text into numeric format. The word2vec model is the most popular method in NLP, since it can give meaningful answers like vector(“woman”) + vector(“king”)—vector(“man”) = vector(“queen”) and the vector representation provided by this method for semantically similar words are close to each other. Doc2vec model provides fixed-length vector representation for a varying length of paragraph so we have used this method to represent varying post length with fixed-length word embeddings. In the following subsections these algorithms are introduced concisely.

### 3.1 Deep neural network (DNN)

**Fig 2:**
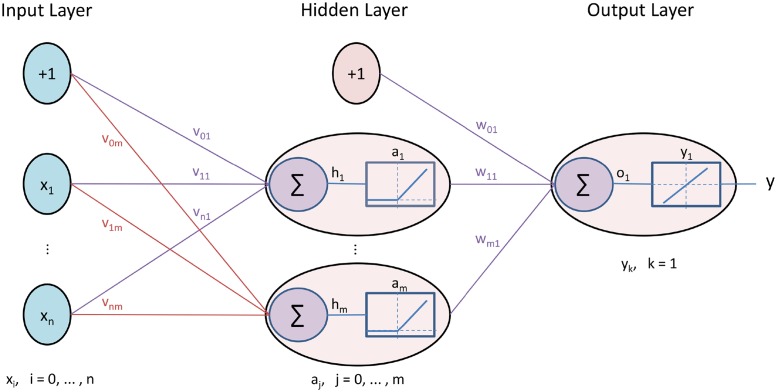
Artificial neural network.

A DNN is an extended term for artificial neural network (ANN) with many hidden layers. A feed-forward neural network consists of an input layer, an output layer and one or more hidden layers. If the number of hidden layers is more than one then it qualifies the term “deep”, hence the name deep neural network [31]. For the sake of simplicity let’s take an example of single hidden layer feed-forward neural network (SLFN) Fig 2 for introductory details of this algorithm which can be further extended to multiple hidden layers.

In the forward pass of neural network training, the inputs *x*_*i*_, where *i* = 1, …, *n* is fed to the input layer of neural network and weighted sum of hidden layer is calculated. A bias *v*_0*j*_, where *j* = 1, …, *m* is also added while calculating weighted sum of hidden layer.

$$\begin{array}{c} h_{j}=v_{i}^{T}x=\sum_{i=1}^{n}v_{ij}x_{i}+v_{0j} \end{array}$$ (1)

Next, this weighted sum is passed through the activation function of hidden layer. In recent years, rectified linear unit (ReLU) is most popular to be used as activation function since it learns faster in multilayer networks [32].

$$\begin{array}{c} a_{j}=ReLU\left(h_{j}\right)=max\left(h_{j},0\right) \end{array}$$ (2)

After the hidden layer output *a*_*j*_, the weighted sum of output layer is calculated with bias as *w*_0*k*_ where k = 1 since only one node is there in output layer.

$$\begin{array}{c} o_{k}=w_{j}^{T}a=\sum_{j=1}^{m}w_{jk}a_{j}+w_{0k} \end{array}$$ (3)

For the final output this weighted sum is passed through the activation function of output layer. In our study, we need to predict the total interaction for a Facebook post which is a regression problem so we used linear activation function in output layer.

$$\begin{array}{c} y_{k}=f\left(o_{k}\right)=o_{k} \end{array}$$ (4)

The mean squared error is calculated between target *t*_*k*_ and predicted output *y*_*k*_. *l* represents the number of nodes in output layer.

$$\begin{array}{c} E\left(t,y\right)=\frac{1}{l}\sum_{k=1}^{l}{\left(t_{k}-y_{k}\right)}^{2} \end{array}$$ (5)

In backward pass, the calculated error is back-propagated using adam optimizer [33] with learning rate of 0.001 to update the weights of neural network model.

### 3.2 Extreme Learning Machine (ELM)

The ELM method is fastest approach to train neural network in comparison to DNN. Instead of using backpropagation where each layer is tuned multiple times, this method uses SLFN where input weights **w**_*i*_ and hidden layer biases *b*_*i*_ are randomly assigned and never updated. The output weights *β*_*i*_ are analytically determined in a single step [21].

Since this is a comparative study so we have used same activation function ReLU in hidden layer as it was used in DNN and the output node is chosen as linear. A SLFN with *L* hidden nodes and activation function *g*(*x*) can be represented as:

$$\begin{array}{c} f_{L}\left(x\right)=\sum_{i=1}^{L}\beta_{i}g\left(w_{i}\cdot x_{j}+b_{i}\right),\;j=1,\ldots ,N \end{array}$$ (6)

where *N* is the number of training samples, **w**_*i*_ = [*w*_1*i*_, *w*_2*i*_, …, *w*_*ni*_]^*T*^ is the weight vector connecting *n* input nodes to *i*th hidden node, **β**_*i*_ = [*β*_11_, *β*_21_, …, *β*_*L*1_] is the weight vector connecting *L* hidden nodes to one output node and *b*_*i*_ is the bias of *i*th hidden node.

From Eq 6, the hidden layer output and final output is given as:

$$\begin{array}{c} h\left(x\right)=\sum_{i=1}^{L}g\left(w_{i}\cdot x_{j}+b_{i}\right) \end{array}$$ (7)

$$\begin{array}{c} y=h(x)\cdot \beta \end{array}$$ (8)

The output weights *β* is unknown but the target values **T** of training samples are known. So the hidden layer output *h*(**x**) and target values **T** can be used to find output weights *β*. After input weights and hidden layer biases are randomly chosen, a SLFN can be considered as a linear system which can be represented as:

$$\begin{array}{c} H\beta =T \end{array}$$ (9)

where the hidden layer output matrix **H** can be written as:

$$\begin{array}{c} \begin{array}{cc} H & =[\begin{array}{c} h(x_{1})\\ h(x_{2})\\ \vdots \\ h(x_{N}) \end{array}]=[\begin{array}{cccc} h_{1}\left(x_{1}\right) & h_{2}\left(x_{1}\right) & \cdots & h_{L}\left(x_{1}\right)\\ h_{1}\left(x_{2}\right) & h_{2}\left(x_{2}\right) & \cdots & h_{L}\left(x_{2}\right)\\ \vdots & \vdots & \ddots & \vdots \\ h_{1}\left(x_{N}\right) & h_{2}\left(x_{N}\right) & \cdots & h_{L}\left(x_{N}\right) \end{array}]\\ & =[\begin{array}{ccc} g(w_{1}\cdot x_{1}+b_{1}) & \cdots & g(w_{L}\cdot x_{1}+b_{L})\\ g(w_{1}\cdot x_{2}+b_{1}) & \cdots & g(w_{L}\cdot x_{2}+b_{L})\\ \vdots & \ddots & \vdots \\ g(w_{1}\cdot x_{N}+b_{1}) & \cdots & g(w_{L}\cdot x_{N}+b_{L}) \end{array}] \end{array} \end{array}$$ (10)

**Fig 3:**
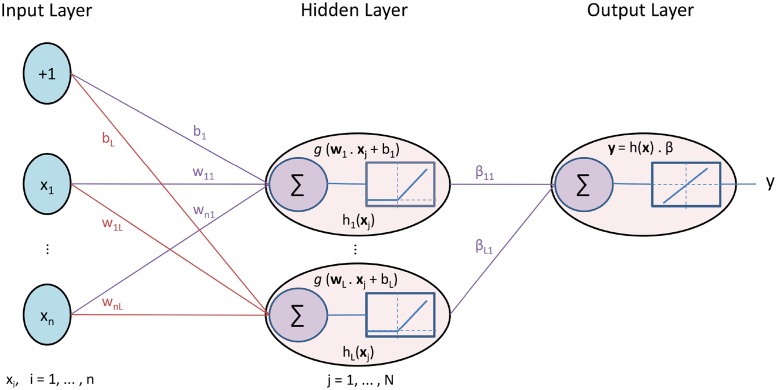
Extreme Learning Machine.

The *i*th column of **H** is the *i*th hidden node output vector with respect to inputs **x**_1_, **x**_2_, …, **x**_*N*_. As from Fig 3, Eqs 7 and 10, it is obvious that the output of each hidden node *g*(**w**_*i*_ ⋅ **x**_*j*_ + *b*_*i*_) is the output from all training samples instead of single sample as was the case in DNN.

So, the output of first hidden node from first and last sample can be expanded as:

$$\begin{array}{cc} h_{1}\left(x_{1}\right) & =g(w_{1}\cdot x_{1}+b_{1})\\ & =g(w_{11}x_{11}+w_{21}x_{21}+\cdots +w_{n1}x_{n1}+b_{1}) \end{array}$$

$$\begin{array}{cc} h_{1}\left(x_{N}\right) & =g(w_{1}\cdot x_{N}+b_{1})\\ & =g(w_{11}x_{1N}+w_{21}x_{2N}+\cdots +w_{n1}x_{nN}+b_{1}) \end{array}$$

where *x*_11_ is the first attribute of first sample, *x*_21_ is the second attribute of first sample and so on.

The target matrix **T** can be written as:

$$\begin{array}{c} T=[\begin{array}{c} t_{1}^{T}\\ t_{2}^{T}\\ \vdots \\ t_{N}^{T} \end{array}]=[\begin{array}{c} t_{11}\\ t_{21}\\ \vdots \\ t_{N1} \end{array}] \end{array}$$ (11)

If the number of training samples *N* and number of hidden nodes *L* are same i.e., *L* = *N*, then **H** will be a square matrix and invertible so output weights *β* can be calculated using *β* = **H**^−1^ **T** which can approximate these training samples with zero error. But in most cases *L* ≪ *N*, then **H** will be a non-square matrix and the output weights can be calculated as:

$$\begin{array}{c} \hat{\beta }=H^{†}T \end{array}$$ (12)

where **H**^†^ is the Moore-Penrose generalized inverse of matrix **H** and **H**^†^ = (**H**^*T*^ **H**)^−1^ **H**^*T*^, if **H**^*T*^ **H** is nonsingular; or **H**^†^ = **H**^*T*^(**H**^*T*^ **H**)^−1^, if **H** **H**^*T*^ is nonsingular.

### 3.3 Long Short-Term Memory (LSTM)

In feed-forward neural networks only current input is considered at any instant of time to predict the output while in recurrent neural networks (RNN), the information obtained in previous timesteps are also considered in output prediction. But as the number of timesteps is increased to look into the information history, it suffers from vanishing gradient problem while backpropagating error through that many timesteps.

**Fig 4:**
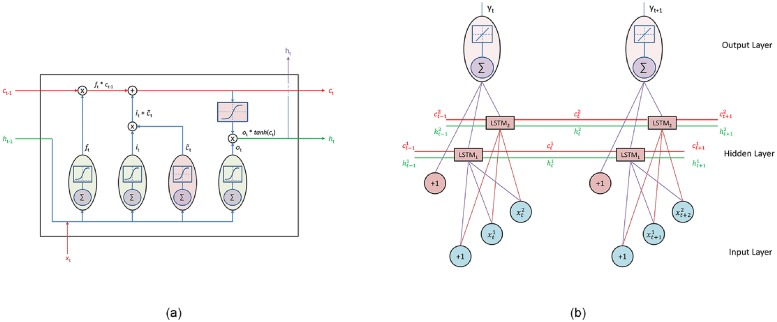
Long Short-Term Memory. (a)LSTM Architecture (b)LSTM Networks.

LSTM is a special kind of RNN which solves this vanishing gradient problem encountered in RNN [22], [23]. For the first input, zero vector is used as previous timesteps information. LSTM incorporates three gates which controls the information of cell state *c*_*t*_, the long-term memory Fig 4(a). The gates activation being sigmoid with [0,1] decides the amount of information to flow through it.

The forget gate decides the amount of information to be removed from the cell state.

$$\begin{array}{c} f_{t}=\sigma \left(W_{fh}h_{t-1}+W_{fx}x_{t}+b_{f}\right) \end{array}$$ (13)

where *x*_*t*_ is the input, *W*_*fx*_ is the weights between input and forget gate of LSTM, *h*_*t*−1_ is previous hidden state value, *W*_*fh*_ is the weight between previous hidden state and forget gate, *b*_*f*_ is the bias for forget gate.

The input gate decides the amount of information to be added to cell state.

$$\begin{array}{c} i_{t}=\sigma \left(W_{ih}h_{t-1}+W_{ix}x_{t}+b_{i}\right) \end{array}$$ (14)

The candidate values ${\tilde{c}}_{t}$ decides the new information to be added to cell state.

$$\begin{array}{c} {\tilde{c}}_{t}=\sigma \left(W_{ch}h_{t-1}+W_{cx}x_{t}+b_{c}\right) \end{array}$$ (15)

The updated cell state *c*_*t*_ is the combination of removing some information from previous cell state *c*_*t*−1_ and adding new candidate values ${\tilde{c}}_{t}$ which is given as:

$$\begin{array}{c} c_{t}=f_{t}*c_{t-1}+i_{t}*{\tilde{c}}_{t} \end{array}$$ (16)

The output gate decides the amount of information to be given out from updated cell state.

$$\begin{array}{c} o_{t}=\sigma \left(W_{oh}h_{t-1}+W_{ox}x_{t}+b_{o}\right) \end{array}$$ (17)

The cell state is squashed between [-1,1] with tanh, before updating the hidden state.

$$\begin{array}{c} h_{t}=o_{t}*tanh\left(c_{t}\right) \end{array}$$ (18)

After the output prediction, mean squared error will be calculated between target and predicted output which will be back-propagated using adam [33] optimizer with a learning rate of 0.001.

### 3.4 Word vectors (word2vec)

**Fig 5:**
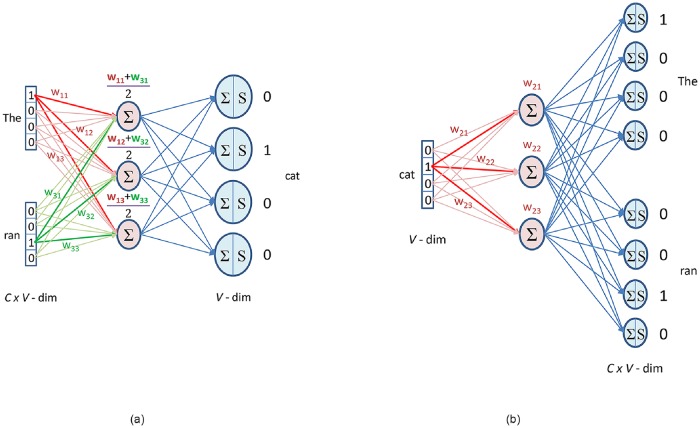
Word vectors. (a)Continuous bag-of-words (b)Skip-gram.

Word2vec has been the most famous NLP technique that provides distributed word representations and the generated vectors have syntactic and semantic word similarities. Two model architectures are proposed for generating word2vec i.e., continuous bag-of-words (CBOW) and skip-gram model. In CBOW model, word2vec for each word is generated using word as the target and neighbouring or context words as input Fig 5(a), while in skip-gram model, it is vice versa Fig 5(b). The CBOW architecture works slightly better than skip-gram in syntactic tasks while skip-gram works better in semantic tasks [24].

The weight matrix between input and hidden layer are the word vectors that we are trying to train using the two model architectures. The pre-trained vectors for 3 million words and phrases were provided by authors of [26] which was trained on about 100 billion words using CBOW architecture. As a part of comparative analysis we have also used these pre-trained word vectors in our study. In word2vec, first a vocabulary of size V is created then each word is represented as one-hot encoded vector with 1 being at the word position in vocabulary and 0 elsewhere. This one-hot encoded vector will be used as a representation of each word in vocabulary.

#### 3.4.1 Continuous bag-of-words model

The CBOW model consists of an input layer with context words, hidden layer with number of nodes as per the word vector dimension and an output layer with target word. The context words are selected as per the window size. If window size is one, then one context word is selected from history and one from future and middle word needs to be predicted at the output layer. Each context words share same weights between input and hidden layer.

For example, if we have a sentence “The cat ran away” and “cat” is the target word then for window size one “The” and “ran” will be the context words. Since we have only four words in our vocabulary so the one-hot encoded vectors for each words are [1, 0, 0, 0], [0, 1, 0, 0], [0, 0, 1, 0] and [0, 0, 0, 1]. From Fig 5(a), “The” contribution to hidden layer is only through the product of 1 to input-hidden weights, rest of the input being 0 has no contribution. Similarly, “ran” contributes through different sets of weights and average of context words contribution is calculated.

$$\begin{array}{c} h=\frac{1}{C}w^{T}\sum_{i=1}^{C}x_{i} \end{array}$$ (19)

where C is the number of context words. Further the product of hidden layer output and hidden-output weight matrix is calculated.

$$\begin{array}{c} u_{j}=v^{T}h,\;j=1,\ldots ,V \end{array}$$ (20)

where V is the size of vocabulary. This output is passed through a softmax function which provides positive probabilities summing to 1.

$$\begin{array}{c} p\left(w_{j}|w_{I}\right)=y_{j}=\frac{e^{u_{j}}}{\sum_{k=1}^{V}e^{u_{k}}} \end{array}$$ (21)

where *w*_*j*_ is the output word, *w*_*I*_ is the input context words and *y*_*j*_ is the output of *j*^*th*^ unit in output layer. The probability distribution at the output layer is compared with one-hot encoded vector of target word *t*_*j*_.

$$\begin{array}{c} e_{j}=y_{j}-t_{j} \end{array}$$ (22)

This error is backpropagated to update the weights [34].

#### 3.4.2 Skip-gram model

The skip-gram model is similar to CBOW model except that the target word is at the input layer and context words are at the output layer. In this model one random word is selected from context words to be predicted at the output layer. Further the probability distribution obtained at the output layer is compared with one-hot encoded vector of all context words and the combined error is backpropagated.

Since input layer contains only one word so the product of input vector and input-hidden weight matrix is directly calculated.

$$\begin{array}{c} h=w^{T}x \end{array}$$ (23)

where *x* is the input vector. Each context words share same weights between hidden and output layer. So the probability distribution obtained at the output layer will be same for all context words. The predicted output is compared with one-hot encoded vector for all context words.

$$\begin{array}{c} e_{c,j}=y_{c,j}-t_{c,j} \end{array}$$ (24)

where *y*_*c*,*j*_ is the predicted output and *t*_*c*,*j*_ is the actual output of *j*^*th*^ unit for context word *c*. At last all the errors of context words are added together.

$$\begin{array}{c} E_{j}=\sum_{c=1}^{C}e_{c,j} \end{array}$$ (25)

The combined error is backpropagated to update the weights.

### 3.5 Paragraph vectors (doc2vec)

Doc2vec is an extension to word2vec where fixed length vector representation is obtained for variable length of text like sentences, paragraphs and documents. Two model architectures are proposed for generating doc2vec i.e., distributed memory model of paragraph vectors (PV-DM) and distributed bag-of-words model of paragraph vectors (PV-DBOW) [25]. The input-hidden weight matrix are the paragraph and words vectors that we are trying to train using the two model architechtures.

#### 3.5.1 PV-DM

**Fig 6:**
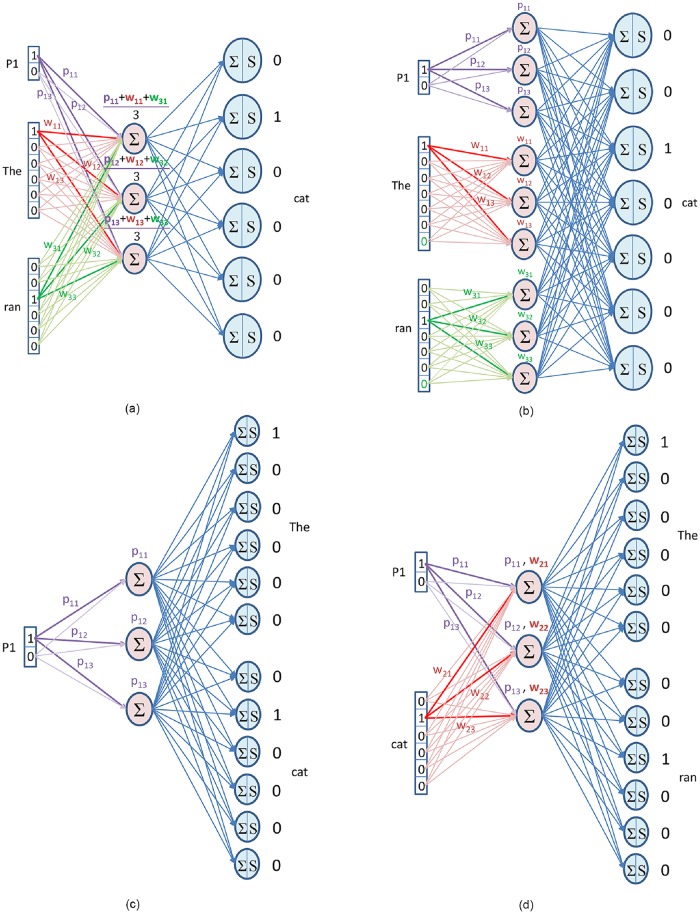
Paragraph vectors. (a)Distributed memory (Mean) (b)Distributed memory (Concatenation) (c)Distributed bag-of-words (d)Distributed bag-of-words with skip-gram.

This model is similar to CBOW model of word2vec except that in this model a token or id is used to represent each paragraph and this paragraph token will also contribute along with context words to predict target word. The paragraph vectors and word vectors are averaged Fig 6(a) or concatenated Fig 6(b) to predict the target word.

Let us take an example of two sentences “The cat ran away” and “The cat was black” and represent both sentences as P1 and P2 respectively. The one-hot encoded vector for each sentence will be [1, 0] and [0, 1]. The vocabulary of unique words in these sentences are [“The”, “cat”, “ran”, “away”, “was”, “black”] and corresponding one-hot encoded vectors are [1, 0, 0, 0, 0, 0], [0, 1, 0, 0, 0, 0], [0, 0, 1, 0, 0, 0] and so on. To find the doc2vec of each sentence, a window size will be selected and this window will slide through each words of the sentence. The context and target words will change as the window slides but the one-hot vector of paragraph will remain the same until the end of sentence. As the window moves to second sentence the one-hot vector of paragraph also changes as per the new sentence. The context words share the same weights so “The” contribution will be through product of 1 and its corresponding weights while “ran” contributes through different sets of weights.

If paragraph and word vectors are averaged to predict the target word, we call this model distributed memory mean (dmm) and if it is concatenated we call it distributed memory concatenation (dmc). In dmc model Fig 6(b), a null word is added in vocabulary with existing words. This is needed because if the target word is first word of the sentence then future words can be selected from paragraph but history words will be selected as null word.

#### 3.5.2 PV-DBOW

The PV-DBOW model is similar to skip-gram model of word2vec except that in this model one-hot encoded vector of paragraph token will be used as input to predict context words which we call distributed bag-of-words (dbow) Fig 6(c). Similar to skip-gram, one random word from context words will be selected to be predicted at the output layer. The output provides a probability distribution which is compared with one-hot vector of context words and combined error is backpropagated to update the weights.

The PV-DBOW implementation in gensim [35] provides an option to train paragraph vectors along with word vectors in the skip-gram fashion which we call distributed bag-of-words with skip-gram (dbow-sg) Fig 6(d). Since the hidden-output weights are shared between word vectors and paragraph vectors so word vectors will influence the paragraph vectors to attain better vector representation of paragraph. This model performed well in [36], so we have included this model in our comparative study.

## 4 Dataset and data preprocessing

Recently e-commerce companies have evolved rapidly which in turn created a massive growth of customers buying products online [37], [38], so we have analysed some e-commerce companies page on Facebook to collect dataset for our study. We analysed the Facebook page for Alibaba, Amazon, Flipkart, Snapdeal and Ebay to find the oldest page with more user interaction. Since the page created date is not available through Facebook Graph API, we checked the first post created date on these pages and found that the first post created on Amazon was on March 28, 2008 which is oldest among all five pages. Since this study is comparative analysis on Facebook post interaction so we need a page with more user interaction which is direct result of more page likes that also belongs to Amazon with 28 million page likes. So the dataset is collected from Amazon Facebook page (https://www.facebook.com/Amazon/).

The dataset is collected for 5 years from October 1, 2012 to September 30, 2017 using Facebook Graph API (https://developers.facebook.com/docs/graph-api/). The posts published by admin or moderator of the page are only considered for the analysis which represents company’s voice while posts by page fans are discarded. This dataset consists of 3,144 published posts. The attributes or features of posts collected are shown in Table 1. It is a well known fact that low quality data leads to low quality knowledge [39], so to improve the data quality, data preprocessing techniques are applied to obtain the final dataset. Data preprocessing helps to remove noise, redundant and irrelevant data. The preprocessing techniques applied to our dataset are dimensionality reduction, instance reduction, feature indexers and noise treatment [40]. The final features after applying data preprocessing techniques are shown in Table 2.

**Table 1:** Features collected from Amazon Facebook page.

Feature	Description	Data type
Message	Post content	Text
Type	Content type (Photo, Video, Link, Status, Offer, Event)	Text
Date	Post created date	Datetime
Likes	Number of likes on post	Numeric
Love	Number of love reaction on post	Numeric
Wow	Number of wow reaction on post	Numeric
Haha	Number of haha reaction on post	Numeric
Sad	Number of sad reaction on post	Numeric
Angry	Number of angry reaction on post	Numeric
Comments	Number of comments on post	Numeric
Shares	Number of shares on post	Numeric
Page total likes	Number of users liked the company page	Numeric

**Table 2:** Features after data preprocessing.

Feature/Label	Description	Data type
Message	Post content	Text
Type	Content type (1, 2, 3, 4, 5, 6)	Numeric
Month	Post published month (1 through 12)	Numeric
Weekday	Post published weekday (1 for Monday to 7 for Sunday)	Numeric
Hour	Post published hour (0 to 23)	Numeric
Duration	Duration between consecutive posts (in seconds)	Numeric
Total interactions	Sum of reactions (Likes, Love, Wow, Haha, Sad, Angry), comments and shares	Numeric

### 4.1 Dimensionality reduction

Dimensionality reduction is applied to remove redundant and irrelevant data. The two ways to apply dimensionality reduction are feature selection and feature extraction [41].

#### 4.1.1 Feature selection

Feature selection is the preprocessing step in which a subset of original features is selected that preserves relevant information [42]. The “page total likes” attribute has same value for each record because the Facebook graph API provides total page likes at the time when data is retrieved so this feature is excluded from the dataset.

#### 4.1.2 Feature extraction

Feature extraction is the preprocessing step in which transformation of original features is done to generate a new set of features which are more significant [42]. Feature extraction is also sometimes referred to as feature construction [43]. The reaction features (“Likes”, “Love”, “Wow”, “Haha”, “Sad”, “Angry”) are helpful for brands to know the emotional responses of users on a post content. These reactions along with user comments is a key measure to know user sentiments towards a post. The more number of post shares meant the more brand reach to more users. The user interaction through reactions, comments and shares can increase brand popularity so these three features are added together to generate a new feature “Total interactions”.

$$\begin{array}{c} \text{Total}\;\text{interactions}=\text{Reactions}+\text{Comments}+\text{Shares} \end{array}$$

where,

$$\begin{array}{c} \text{Reactions}=\text{Likes}+\text{Love}+\text{Wow}+\text{Haha}+\text{Sad}+\text{Angry} \end{array}$$

The new features “Month”, “Weekday” and “Hour” are extracted from “Date”. The initial features through which the new features are generated are removed after feature extraction. A new feature “Duration” is also extracted from “Date” after noise treatment which provides duration between posts.

### 4.2 Instance reduction

Instance reduction is to samples of data as dimensionality reduction is to features of data. It reduces the quantity of data by removing some samples of data which is called instance selection or generating new ones which is instance generation. Almost all samples in dataset has unique Uniform Resource Locator (URL) in post content. Since each URL is unique so generating word embeddings for these URLs will increase the vocabulary size but it will be less efficient because embeddings generated for these URLs will have insignificant meaning. So we discarded the URLs while generating word embeddings. There are five samples which contains only URLs in “Message”. Since URLs are discarded from the “Message”, so post content will be empty for these five samples. To avoid this situation, instance selection is used to discard these five samples from dataset which decreases the sample size of dataset from 3,144 to 3,139.

### 4.3 Feature indexers

Feature indexers convert data type of a feature using indexer techniques [40]. String indexer is used to convert data type of “Type” feature from text to numeric. The content type “photo”, “video”, “link”, “status”, “offer” and “event” are changed to 1, 2, 3, 4, 5 and 6 respectively.

### 4.4 Noise treatment

Noise treatment is data preprocessing technique which is used to remove noisy data from dataset. After thorough analysis of the dataset, it is found that the “Total interactions” feature has inconsistency in its values. Some of its values are too large in comparison to other samples. For example, the maximum value for total interaction is found to be 1,30,670 with content type as “video” but none of the previous or next five posts with same content type crossed even 10,000 interactions. We assume that these posts are promoted posts via boosting [44], a paid service of Facebook to increase visibility and engagement for a particular post. These total interaction values being much larger in comparison to other data makes them outliers. Outliers are those values which are too large or too small in comparison to vast majority of observations. These outliers act as noise and it can bias the output of machine learning models. This makes noise treatment necessary to remove the outliers from the dataset.

#### 4.1.1. Mean plus or minus 3 standard deviations

This is the most common method to remove outliers from the dataset. In this method, the values greater or smaller than three standard deviations from mean are eliminated. The inference behind this is that 99.87% of data lies within three standard deviations and outliers lies farther away.

$$\begin{array}{c} \mu -3\sigma <x_{i}<\mu +3\sigma \end{array}$$

where *x*_*i*_ is the majority of data that lies inside three standard deviations. So removing 0.13% of data outside this range does not seems to cause much effect.

#### 4.4.2 Median plus or minus 3 median absolute deviations

Leys et al. [45] suggested an alternative approach of median absolute deviation (MAD) for outliers removal.

$$\begin{array}{c} median-3\cdot MAD<x_{i}<median+3\cdot MAD \end{array}$$

They argued that 3 MAD is a better approach to remove outliers in comparison to 3 standard deviations so we have used 3 MAD approach to exclude boosted posts from our dataset. After removing the outliers, the dataset size is further decreased from 3,139 to 2,457. Out of 2,457 samples, 70% of data i.e., 1,720 samples will be randomly selected to train the model and rest 30% i.e., 737 samples will be used for testing.

As mentioned in Section 4.1.2, after outliers removal a new feature “Duration” is extracted from “Date” which provides the duration between posts. Table 2 displays the final features after data preprocessing.

## 5 Experiments

**Fig 7:**
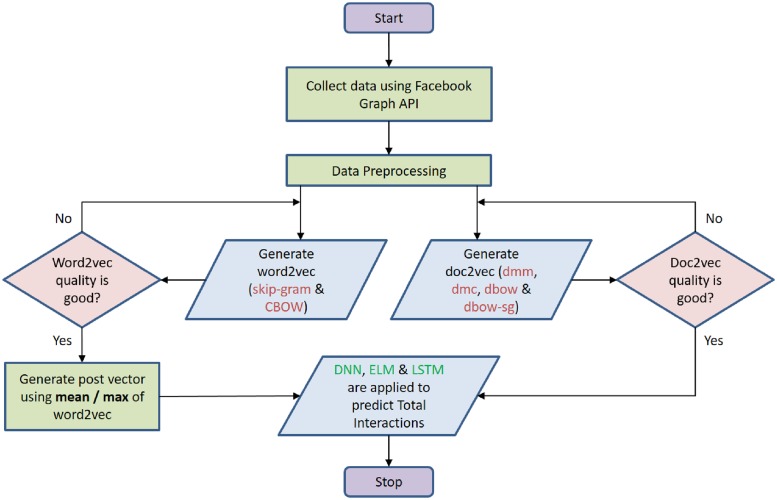
Experiment steps.

The steps involved in our experiment to predict post interaction is shown in Fig 7. After data collection and preprocessing, next step is to create word vectors (word2vec) and paragraph vectors (doc2vec) for post message which can be further used in machine learning models. Each words in word2vec and each posts in doc2vec is represented as 300 dimensional vectors. The quality of word2vec and doc2vec is checked via most similar word and most similar paragraph/post respectively. If the vector quality is not good, then number of epochs are varied to improve the quality. The post vector is generated from word2vec by calculating mean or maximum of all words in a post. These post vectors with 300 features are combined together with remaining five features type, month, weekday, hour and duration. These 305 features are normalized for train and test dataset and further selected as input to predict total interactions using DNN, ELM and LSTM.

### 5.1 Generate post vector using word2vec and doc2vec

The word2vec and doc2vec hidden layer contains 300 nodes which determines the dimension of vector representation. Word2vec provides vector representaion of words which is further used to generate vector representation of each post. The initial learning rate to train word2vec model is selected as 0.025 which is gradually decreased to 0.0001, as training progresses. The mean and maximum of all words vector in a particular post is calculated to generate post vector. While creating word2vec from our dataset, we have used all the post messages since the dataset size is small. The reason behind this is that if only training samples are used to generate word2vec then there will be many words in test data whose vector representation will not be found in training data and discarding those many words will decrease the quality of post vector. The word2vec quality is checked by finding similar words and the best result is obtained with training epochs of 100 as shown in Table 3. There are some words in our dataset whose vector representation is not available in pre-trained vectors provided by Mikolov et al. [26] which we call “google word2vec”, so while generating post vectors those words are discarded.

**Table 3:** Most similar word from word vectors.

Input	Skip-gram			CBOW			Mikolov et al. [24], [26]
	10 epochs	50 epochs	100 epochs	10 epochs	50 epochs	100 epochs	
Gift	Card	Cards	Card	Cards	Cards	Card	gift
Shop	here	furious	Fan	mug	store	shop	Store
Movies	Dot	Shows	Shows	Android	TV	Shows	Movie
Android	Appstore	Appstore	Appstore	eligible	Appstore	Appstore	smartphone
Hurricane	senators	Relief	Relief	cheese	Harvey	Harvey	hurricanes

The doc2vec model is trained to obtain post vector using all the four architectures dmm, dmc, dbow and dbow-sg as mentioned in Section 3.5. The initial learning rate to train doc2vec model is selected as 0.025 which is gradually decreased to 0.001, as training progresses. We have used only training data i.e., 1,720 samples from our dataset to train doc2vec model. The generated post vectors will be used while training machine learning models but in test phase, inference step is performed to obtain post vector. In the inference step, the doc2vec is generated for a new post in a similar way as the training process except that word vectors and output weights are kept constant. The doc2vec quality is also checked by finding similar posts and the best result is obtained with training epochs of 500 as shown in Table 4. Increasing number of epochs further did not increase the post vector quality, so the paragraph vectors obtained with 500 epochs is used in machine learning models.

**Table 4:** Most similar paragraph from paragraph vectors.

Input		Coffee or tea?	How do you find new books to read?	Happy Earth Day!	What’s your favorite way to work out?
**dbow**	100 epochs	Lime Punch or Flame Scarlet? …	How do you find great books to read? …	Happy Middle Earth Day! …	What’s your favorite holiday tale?
	500 epochs	Coffee is magic …	How do you find great books to read? …	Happy Middle Earth Day! …	What’s your favorite winter feast?
**dbow-sg**	100 epochs	Coffee is magic …	How do you find great books to read? …	Happy Middle Earth Day! …	What’s your favorite holiday tale?
	500 epochs	Coffee is magic …	How do you find great books to read? …	Happy Middle Earth Day! …	What’s your favorite holiday tale?
**dmm**	100 epochs	Lime Punch or Flame Scarlet? …	#BeTransparent	Happy Middle Earth Day! …	Easter’s right around the corner! …
	500 epochs	Coffee is magic …	Don’t miss any detail …	Happy Middle Earth Day! …	What’s your favorite winter feast?
**dmc**	100 epochs	Space travel or vampires?	A new Amazon Books store just opened in New York …	…It’s a Prime, Prime Day. Amazon Prime Prime Day	What’s your favorite song of all time? …
	500 epochs	Space travel or vampires?	This box is part of the up to 50% off Gold Box Deal …	Prime Day’s back! …	Today’s Deal of the Day. For him, for her …

### 5.2 Implement post vector in machine learning models

There are six models from word2vec i.e., skip-gram (mean), skip-gram (max), CBOW (mean), CBOW (max), google word2vec (mean), google word2vec (max) and four models from doc2vec i.e., dmc, dmm, dbow, dbow-sg. For single hidden layer, the neurons are varied from 10 to 100 with a gap of 10 i.e., 10, 20, …, 100 and for multiple hidden layers, number of layers are varied from 1 to 5 with 10 neurons in each layer. For each individual models, batch size is considered as 8, 16, 32 and 64. Further epochs size were varied to predict the total interactions with least error.

The root mean square error (*RMSE*) and coefficient of determination (*R*^2^) are used to evaluate the performance of machine learning models.

$$\begin{array}{c} RMSE=\sqrt{\frac{1}{n}\sum_{i=1}^{n}{\left(y_{i}-\hat{y_{i}}\right)}^{2}} \end{array}$$ (26)

$$\begin{array}{c} R^{2}=1-\frac{\sum_{i=1}^{n}{\left(y_{i}-\hat{y_{i}}\right)}^{2}}{\sum_{i=1}^{n}{\left(y_{i}-\bar{y}\right)}^{2}} \end{array}$$ (27)

where *n* is the number of test samples, *y*_*i*_ is the actual total interaction, $\hat{y_{i}}$ is the predicted interaction and $\bar{y}$ is the mean of actual interactions. *RMSE* tells how spread out the prediction errors are and *R*^2^ provides a measure of how well the observed outcomes are replicated by the model. *R*^2^ values lies in the range from 0 to 1 which determines the performance of the model. It can also provide negative values which means prediction is worse than the mean value of actual interactions.

## 6 Results

The performance of word2vec and doc2vec is evaluated by plotting a graph between *R*^2^ and number of neurons in hidden layer for single hidden layer and number of layers for multiple hidden layer. ELM being a single hidden layer architecture, it is not incorporated in multiple hidden layer analysis.

### 6.1 Model analysis: Single hidden layer (word2vec)

**Fig 8:**
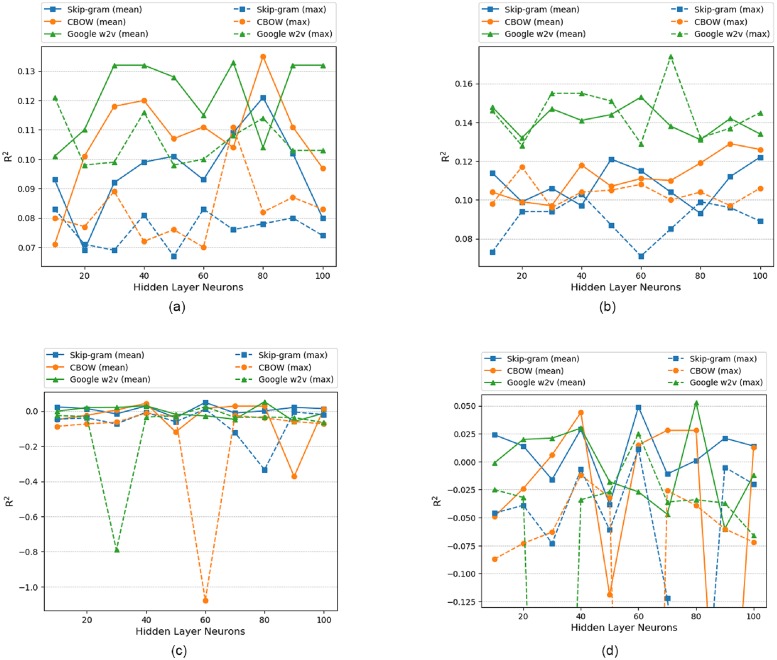
Single hidden layer (Skip-gram vs CBOW vs Google w2v) for ANN, LSTM and ELM. (a)ANN (word2vec) (b)LSTM (word2vec) (c)ELM (word2vec) (d)ELM Rescaled (word2vec).

Fig 8(a), depicts the performance of word2vec in ANN. Google w2v performed well initially but CBOW (mean) reached a maximum *R*^2^ of 0.135 with 80 neurons in hidden layer. The order of performance for all word2vec models are shown in Table 5. Since the *R*^2^ value for skip-gram (mean) and google w2v (max) are same so, the model performance is measured with *RMSE* value which are 771.766 and 772.231 respectively.

**Table 5:** Word2vec order of performance in single hidden layer for ANN, LSTM and ELM.

ANN				LSTM				ELM			
Word2vec order of performance		*R*^2^ (max)	# of Neurons	Word2vec order of performance		*R*^2^ (max)	# of Neurons	Word2vec order of performance		*R*^2^ (max)	# of Neurons
CBOW	mean	0.135	80	Google w2v	max	0.174	70	Google w2v	mean	0.053	80
Google w2v	mean	0.133	70	Google w2v	mean	0.153	60	Skip-gram	mean	0.049	60
Skip-gram	mean	0.121	80	CBOW	mean	0.129	90	CBOW	mean	0.044	40
Google w2v	max	0.121	10	Skip-gram	mean	0.122	100	Google w2v	max	0.025	60
CBOW	max	0.111	70	CBOW	max	0.117	20	Skip-gram	max	0.011	60
Skip-gram	max	0.083	60	Skip-gram	max	0.103	40	CBOW	max	-0.012	40

The question arises here why CBOW (mean) performed best in comparison to other models. As mentioned in [24], CBOW architecture works better on syntactic tasks and our problem is a syntactic problem where each post words combined together has a certain meaning and its vector representation combined with other 5 inputs performed well in predicting total interactions. This analysis is also validated by google w2v (mean) which was also trained on CBOW architecture. The reason behind mean performance better than max is that max may contain vector representation of just few words instead of all the words in a post.

As shown in Fig 8(b), google w2v performed best in LSTM. It is also observed that peak value of *R*^2^ for all models in LSTM except CBOW (mean) performed better than their outcomes in ANN because in LSTM along with current input, previous input information is also preserved to predict total interaction. The quality of google w2v is better which is evident by the output in Table 3 since it was trained on 100 billion words and the presence of previous input information might be the reason for its best performance in LSTM.

The performance of word2vec model in ELM is shown in Fig 8(c). Since the data points are crowded in between *R*^2^ of -0.13 and 0.06 so this region is rescaled in Fig 8(d) to visualize the details. Google w2v (mean) performed well with *R*^2^ of 0.053 at 80 neurons. ELM performed worse in comparison to ANN and LSTM.

### 6.2 Model analysis: Single hidden layer (doc2vec)

**Fig 9:**
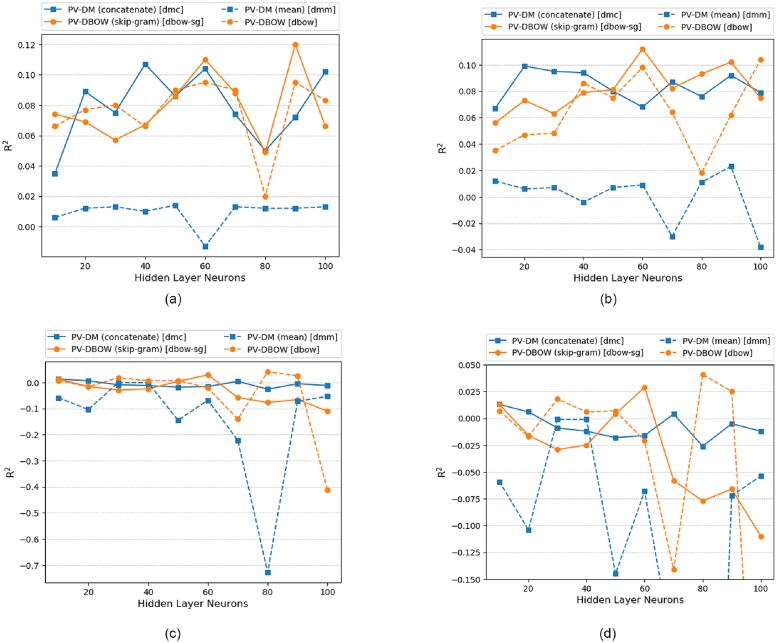
Single hidden layer (PV-DM vs PV-DBOW) for ANN, LSTM and ELM. (a)ANN (doc2vec) (b)LSTM (doc2vec) (c)ELM (doc2vec) (d)ELM Rescaled (doc2vec).

The doc2vec performance in ANN is shown in Fig 9(a). It is found that dbow-sg performed best in prediction task with *R*^2^ of 0.12 at 90 neurons. The reason for dbow-sg model best performance lies in its architecture which was trained in skip-gram fashion of word2vec. The word vectors in dbow-sg influenced paragraph vectors to attain better representation of post. The next order of performance is shown in Table 6. The dmm model performed worse whose maximum *R*^2^ of 0.014 at 50 neurons is lower than the minimum *R*^2^ of all other models in ANN.

**Table 6:** Doc2vec order of performance in single hidden layer for ANN, LSTM and ELM.

ANN			LSTM			ELM		
Doc2vec order of performance	*R*^2^ (max)	# of Neurons	Doc2vec order of performance	*R*^2^ (max)	# of Neurons	Doc2vec order of performance	*R*^2^ (max)	# of Neurons
dbow-sg	0.12	90	dbow-sg	0.112	60	dbow	0.041	80
dmc	0.107	40	dbow	0.104	100	dbow-sg	0.013	10
dbow	0.095	60	dmc	0.099	20	dmc	0.013	10
dmm	0.014	50	dmm	0.023	90	dmm	-0.001	40

In LSTM, dbow-sg again performed best with *R*^2^ of 0.112 at 60 neurons as shown in Fig 9(b). The observations of LSTM shows that PV-DBOW performed better than PV-DM and the reason behind this is that the quality of PV-DBOW vectors are better which is manifested by the paragraph similarity check in Table 4. Also LSTM prediction involves previous input information along with current input which might be the reason for its better performance. It is also found that the performance of dbow and dmm is increased from ANN while performance of dbow-sg and dmc is decreased.

Fig 9(c), shows doc2vec performance in ELM. Since the data points are crowded in between *R*^2^ of -0.15 and 0.05 so this region is rescaled in Fig 9(d), and it is found that dbow performed best in prediction with *R*^2^ of 0.041 at 80 neurons. Since the *R*^2^ value of dbow-sg and dmc are same so, the order of performance is measured with RMSE value which are 817.68 and 817.802 respectively. PV-DBOW again performed better than PV-DM since the quality of post vectors from PV-DBOW are better than PV-DM. ELM again performed worse in doc2vec against ANN and LSTM.

### 6.3 Model analysis: Multiple hidden layers (word2vec)

**Fig 10:**
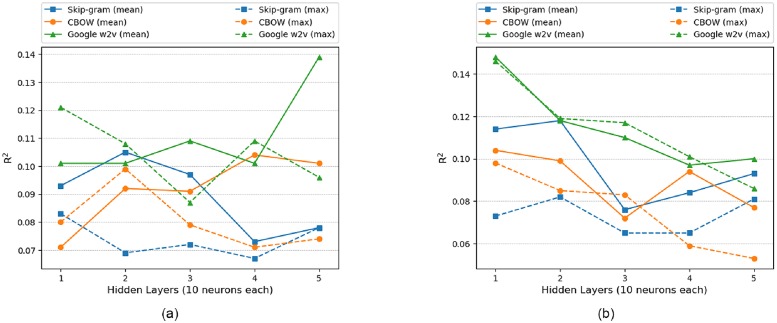
Multiple hidden layers (Skip-gram vs CBOW vs Google w2v) for DNN and LSTM. (a)DNN (word2vec) (b)LSTM (word2vec).

The performance of word2vec in DNN is depicted in Fig 10(a) and Table 7. Due to the better quality of google w2v, it performed best in total interaction prediction. By comparing the results of multiple hidden layers for DNN and single hidden layer of ANN, it is observed that google w2v (max) and skip-gram (max) has same *R*^2^ value of 0.121 and 0.083 respectively as shown in Tables 5 and 7 but the *R*^2^ value for multiple hidden layers came out to be the best when number of layer is one. So it is concluded that single hidden layer performed well in all word2vec models considering ANN and DNN, except google w2v (mean) which provided best performance with *R*^2^ of 0.139 at 5 hidden layers.

**Table 7:** Word2vec order of performance in multiple hidden layers for DNN and LSTM.

DNN				LSTM			
Word2vec order of performance		*R*^2^ (max)	# of Layers	Word2vec order of performance		*R*^2^ (max)	# of Layers
Google w2v	mean	0.139	5	Google w2v	mean	0.148	1
Google w2v	max	0.121	1	Google w2v	max	0.146	1
Skip-gram	mean	0.105	2	Skip-gram	mean	0.118	2
CBOW	mean	0.104	4	CBOW	mean	0.104	1
CBOW	max	0.099	2	CBOW	max	0.098	1
Skip-gram	max	0.083	1	Skip-gram	max	0.082	2

Google w2v again performed best in LSTM as shown in Fig 10(b). It is evident from Table 7 that adding multiple hidden layers in LSTM did not increase the performance of total interaction prediction. The best performance is achieved with either one or two hidden layers. By comparing the results of multiple hidden layers and single hidden layer of LSTM, it is observed that single hidden layer performed best in all word2vec models. The best result of LSTM using word2vec is achieved with *R*^2^ of 0.174 at 70 neurons for google w2v (max) in single hidden layer. One reason behind the poor performance of multiple hidden layers in LSTM is that it needs lots of parameters to be trained since each node in hidden layer contains four gates which are connected to each node in previous layer and also each node contains a recurrent connection that needs to be trained. The small dataset size might be another reason for not achieving better performance by adding multiple hidden layers.

### 6.4 Model analysis: Multiple hidden layers (doc2vec)

**Fig 11:**
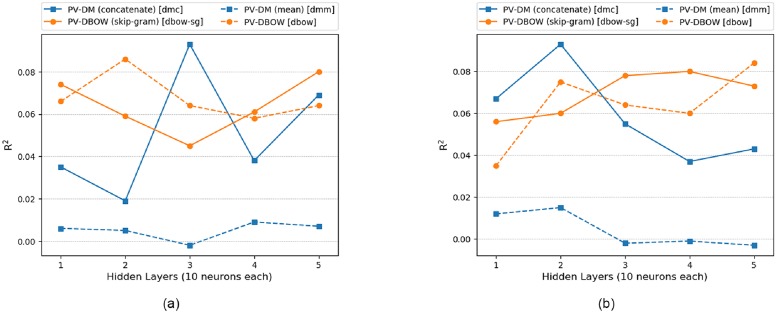
Multiple hidden layers (PV-DM vs PV-DBOW) for DNN and LSTM. (a) DNN (doc2vec) (b)LSTM (doc2vec).

Fig 11(a), depicts doc2vec performance in DNN. The dmc model performed best in total interaction prediction with *R*^2^ of 0.093 at 3 hidden layers, Table 8. The dmm model performed worse and PV-DBOW performance lies in between dmc and dmm. By comparing these results with single hidden layer of ANN, it is observed that single hidden layer performed best in all doc2vec models.

**Table 8:** Doc2vec order of performance in multiple hidden layers for DNN and LSTM.

DNN			LSTM		
Doc2vec order of performance	*R*^2^ (max)	# of Layers	Doc2vec order of performance	*R*^2^ (max)	# of Layers
dmc	0.093	3	dmc	0.093	2
dbow	0.086	2	dbow	0.084	5
dbow-sg	0.08	5	dbow-sg	0.08	4
dmm	0.009	4	dmm	0.015	2

In LSTM, dmc model performed best with *R*^2^ of 0.093 at 2 hidden layers as shown in Fig 11(b). Similar to DNN, dmm performed worse and PV-DBOW performance lies in between dmc and dmm. By comparing these results with single hidden layer of LSTM, it is observed that single hidden layer performed best in all doc2vec models.

### 6.5 Comparative analysis: Word2vec and doc2vec

The above analysis suggests that in DNN and LSTM single hidden layer performed best for word2vec and doc2vec models except google w2v (mean) which performed best with multiple hidden layers in DNN. It is also found that for single hidden layer in ANN, word2vec performed better than doc2vec except CBOW (max) and skip-gram (max). So, it can be said that word2vec (mean) performed better than doc2vec models in ANN. Comparing word2vec and doc2vec models for LSTM in single hidden layer, it is found that word2vec performed better than doc2vec except skip-gram (max). So, word2vec (mean) is better than doc2vec models in LSTM. Comparing word2vec and doc2vec for ELM, it is again found that word2vec (mean) is better than doc2vec. So, it is concluded that word2vec (mean) always performed better than doc2vec models.

The reason for word2vec best performance lies in its model architecture. In word2vec model each word is represented through different sets of weights and the number of times same word is encountered in whole dataset, the weights will be updated that many times for that particular word. In doc2vec model each paragraph is also represented through different sets of weights but the weights related to that paragraph will be updated until the window slides in this paragraph. As the window moves to next paragraph and if the words encountered in first paragraph appears again in next paragraph then it will not contribute to update the weights related to the first paragraph. The weights related to first paragraph will be updated only in next epoch. This might be the reason for poor performance of doc2vec model.

Further analysis from Tables 5 and 7 shows that pre-trained vectors (google w2v) provided best performance in LSTM with single hidden layer to predict Facebook post interaction. The pre-trained google w2v (max) was trained using CBOW architecture with learning rate 0.025 and gradually decreased to zero at the end of training [24]. Further LSTM Fig 4(b), with 70 nodes in hidden layer provided best result to predict Facebook post interaction which was trained with 90 number of epochs and batch size of 32. An adam optimizer is used to train LSTM with learning rate of 0.001. If only those word2vec models that are trained on our dataset are considered then CBOW (mean) performed best in ANN and LSTM with single hidden layer but for multiple hidden layers, skip-gram (mean) performed best in DNN and LSTM.

It is also found that the training time for ELM is lowest since its input weights and hidden layer biases are randomly assigned and output weights are analytically calculated. DNN comes after ELM in training time since it uses backpropagation algorithm to train its synaptic weights in feed-forward neural network. LSTM takes longest time to train since it needs to train its recurrent connection along with feed-forward connection. The order of algorithms as per training time are ELM < DNN < LSTM.

## 7 Conclusion

In this paper, we investigated word2vec and doc2vec performance in prediction of Facebook post interaction using DNN, LSTM and ELM for Amazon Facebook page. Word2vec performed better than doc2vec and CBOW architecture worked better than skip-gram model. It is also found that among DNN, LSTM and ELM, LSTM performed best in prediction, DNN performance comes next and ELM performed worse. Adding multiple hidden layers does not improve LSTM performance but it improved a little bit of performance in DNN. There are certain limitations in this predictive analysis. The complete dataset is used to generate word2vec since the dataset size is small. If only training dataset would have been used, then there will be many words in test set whose vector representation will not be found in training set and ignoring those many words while generating post vector would have decreased the overall performance. This might be one of the reason for better performance of word2vec than doc2vec.

This study can be highly beneficial for companies to predict the impact of post beforehand and make a sound decision about its content and publish time. As per our comparative analysis, LSTM using word embeddings from word2vec provides best approximation about number of user interactions with the post. By varying post content and its published time, the proposed model can be used to increase user interactions with the post which in turn helps in brand popularity to a large customer base.

In future studies, glove [46] and fasttext [47] word vectors can be investigated in comparative analysis and hidden layer nodes in DNN and LSTM can be varied for further analysis. Hierarchical ELM (H-ELM) [48] can be incorporated to analyse its performance by adding multiple hidden layers. Also posts from multiple e-commerce companies can be incorporated in future study with page total likes as an additional feature which will make the model more generic to predict total interactions.
